# Candidate Genes Associated with Abiotic Stress Response in Plants as Tools to Engineer Tolerance to Drought, Salinity and Extreme Temperatures in Wheat: An Overview

**DOI:** 10.3390/plants11233358

**Published:** 2022-12-02

**Authors:** Daniela Trono, Nicola Pecchioni

**Affiliations:** Consiglio per la Ricerca in Agricoltura e l’Analisi dell’Economia Agraria, Centro di Ricerca Cerealicoltura e Colture Industriali, S.S. 673, Km 25,200, 71122 Foggia, Italy

**Keywords:** wheat, abiotic stresses, drought, salinity, cold, heat, genetic engineering, genome editing, transgenesis

## Abstract

Wheat represents one of the most important staple food crops worldwide and its genetic improvement is fundamental to meeting the global demand of the growing population. However, the environmental stresses, worsened by climate change, and the increasing deterioration of arable land make it very difficult to fulfil this demand. In light of this, the tolerance of wheat to abiotic stresses has become a key objective of genetic improvement, as an effective strategy to ensure high yields without increasing the cultivated land. Genetic erosion related to modern agriculture, whereby elite, high-yielding wheat varieties are the product of high selection pressure, has reduced the overall genetic diversity, including the allelic diversity of genes that could be advantageous for adaptation to adverse environmental conditions. This makes traditional breeding a less effective or slower approach to generating new stress-tolerant wheat varieties. Either mining for the diversity of not-adapted large germplasm pools, or generating new diversity, are the mainstream approaches to be pursued. The advent of genetic engineering has opened the possibility to create new plant variability and its application has provided a strong complement to traditional breeding. Genetic engineering strategies such as transgenesis and genome editing have then provided the opportunity to improve environmental tolerance traits of agronomic importance in cultivated species. As for wheat, several laboratories worldwide have successfully produced transgenic wheat lines with enhanced tolerance to abiotic stresses, and, more recently, significant improvements in the CRISPR/Cas9 tools available for targeted variations within the wheat genome have been achieved. In light of this, the present review aims to provide successful examples of genetic engineering applications for the improvement of wheat adaptation to drought, salinity and extreme temperatures, which represent the most frequent and most severe events causing the greatest losses in wheat production worldwide.

## 1. Introduction

Wheat is one of the most important food crops widely cultivated in the world with a production of about 780 million tons on an area of 215 million hectares [1]. In addition to being an important source of dietary calories and proteins for about one-third of the world’s population, wheat grains also provide minerals, dietary fibres, antioxidants and vitamins, which have recognized health benefits since they reduce the risk of cancer, cardiovascular diseases, obesity, type 2 diabetes and other chronic diseases [2]. According to the FAO’s long-term projections towards 2050, the demand for wheat could reach 900 million tons [3]. To meet this global demand, wheat production must be increased by approximately 77% of existing production [4]. Fulfilling this demand is very challenging in view of the current declining of arable land and climate change. An effective strategy to achieve this goal without increasing the area of cultivated land is to improve the tolerance of wheat to environmental stresses in order to ensure yield stability even under adverse climate conditions. With this goal in mind, breeders have turned their efforts towards the selection of key traits driving wheat yield adaptation to environmental challenges, such as drought, salinity, or extreme temperatures, for the development of resilient cultivars.

Domestication, green revolution, and modern plant breeding have led to a significant loss of genetic diversity in wheat. In particular, modern cultivars are less tolerant to both biotic and abiotic stresses than their wild relatives due to the erosion of useful genes that occurred in the course of the selection for high-yielding genotypes in fertile environments [5]. This narrowing of the genetic base restricts the future improvement of wheat for higher tolerance to environmental constraints through the traditional breeding approaches, which rely on the existence of genetic diversity to identify contrasting parents for desired traits to be crossed for the creation of new improved genotypes. One of the future strategies to overcome the loss of tolerance genes by genetic erosion is the use of recombinant DNA technology combined with transgenesis, which allows the transfer of genes from wild relatives or other species. Transgenesis consists of the delivery of a foreign gene into the plant cell, thus obtaining a stable integration of the gene into the nuclear genome; then, fertile plants are regenerated from the transformed cells and the expression of the introduced gene is verified. Over the last two decades, significant achievements have been made to develop a variety of efficient transformation methods in plants; among these, particle bombardment-mediated transformation and *Agrobacterium tumefaciens* systems have been successfully used for the genetic transformation of wheat. A detailed treatment of this topic is beyond the scope of this review, and the reader is referred to excellent reviews that have described in detail the progress made towards the development of robust systems for the genetic transformation of wheat [6,7].

During the last decade, genome-editing technologies have emerged as a powerful tool for crop improvement. Genome-editing tools use engineered nucleases that trigger a double-strand break (DSB) at a desired genomic location. Recently, the possibility of generating targeted DSB has been greatly simplified by the discovery of the type II Clustered Regularly Interspaced Short Palindromic Repeat (CRISPR) system, which uses a specific RNA sequence called ‘single guide RNA’ (sgRNA) to recognize the target region of interest and direct the nuclease there for editing [8]. The DSB is converted into a mutation (insertion or deletion) by the cellular repair machinery through an imprecise, non-homologous end joining, which results in the loss of function of the targeted gene. Precise changes in the desired gene are also possible through homology-dependent repair. In this case, a ‘donor DNA’ containing the desired mutation is introduced in the cell together with the sgRNA and the nuclease; the cellular repair machinery will use the donor DNA to repair the DSB and the mutation will be incorporated in the targeted gene. Although transgenesis and genome editing are both based on plant transformation, these two approaches differ greatly from each other. Transgenesis yields random insertions of foreign genes in the genome of the recipient plant, while genome editing introduces targeted modifications in endogenous genes, thus generating genome-edited lines free of foreign genetic elements, that are therefore identical to their non-transformed counterpart. This overcomes many of the issues associated with transgenesis and increases the probability of public acceptance of transgene-free food crops developed by genome editing. For readers interested in the development of highly efficient tools for the application of genome editing in wheat breeding, we suggest they refer to recent papers on this topic [9,10,11].

Unlike traditional breeding, crop improvement using transgenesis or genome editing requires the identification and validation of candidate genes responsible for the control of the trait to be improved; therefore, the identification and validation of candidate genes associated with wheat tolerance to abiotic stresses are essential in such approaches. Studies on model plants have contributed to the identification of several candidate genes underlying tolerance to abiotic stresses and the orthologous of some of these genes have been identified in crops including wheat. This review provides examples of how some of these abiotic stress-related genes have been successfully used in the genetic engineering of wheat for the improvement of tolerance to drought, salinity and high and low temperatures, which represent the most frequent and most severe events causing the greatest losses in wheat production worldwide.

## 2. Candidate Genes

**Figure 1:**
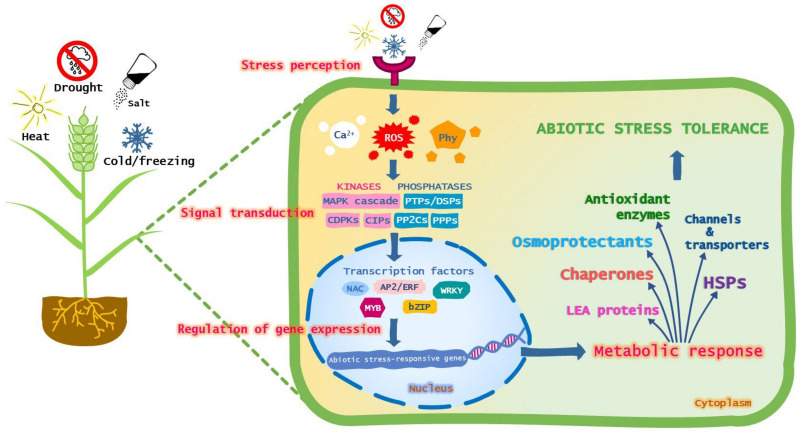
Schematic representation of the signalling pathway leading to the plant response to abiotic stresses. Specific receptors in the plasma membrane perceive the external stress signal and transmit the signal intracellularly through phytohormones and secondary messengers, such as calcium (Ca^2+^) and reactive oxygen species (ROS). The second messengers activate different classes of protein kinases, including mitogen-activated protein kinase (MAPK) cascade, calcium-dependent protein kinases (CDPKs), and calcineurin-B-like proteins-interacting protein kinases (CIPKs), and protein phosphatases, such as protein tyrosine phosphatases/dual-specificity phosphatases (PTPs/DSPs), protein phosphatases 2C (PP2Cs), and serine/threonine-specific protein phosphatases (PPPs). Subsequently, the protein kinases and phosphatases catalyze the phosphorylation/dephosphorylation of transcription factors, including APETALA2/ethylene response element-binding factors (AP2/ERF), the large NAC family, basic leucine zipper (bZIP), WRKY, and MYB. These finally regulate the expression of abiotic stress-responsive genes encoding heat shock proteins (HSPs) and other chaperones, late embryogenesis abundant (LEA) proteins, enzymes involved in the biosynthesis of osmolytes, antioxidant enzymes and enzymes involved in the biosynthesis of small antioxidant molecules, aquaporins and ion transporters, which contribute to the tolerance of wheat to abiotic stresses.

When the biochemical pathway related to a trait of interest is well known, the candidate genes may be chosen among the key genes that regulate that pathway. It is well known that abiotic stresses such as drought, salinity, heat and cold stress, even if very different, cause similar disorders inside the plant cell, including membrane damage, oxidative damage, protein denaturation and osmotic stress, and lead to the activation of a common network of pathways that helps plants to survive. As a general scenario, the stress signal is perceived by specific receptors located in the plasma membrane (Figure 1). After recognition, the extracellular signal is transduced into the cell through phytohormones and second messengers including reactive oxygen species (ROS) and calcium ions (Figure 1). These activate the phosphorylation/dephosphorylation cascade mediated by protein kinases and phosphatases (Figure 1). The final response is the phosphorylation/dephosphorylation-dependent activation/suppression of transcription factors, which can in turn regulate the expression of abiotic stress-responsive genes (Figure 1). These mainly include genes encoding (i) proteins that function directly in the protection of proteins and membranes, such as late embryogenesis abundant (LEA) proteins, heat shock proteins (HSPs) and other chaperones, (ii) enzymes that are involved in the biosynthesis of osmolytes, (iii) antioxidant enzymes and enzymes involved in the biosynthesis of small antioxidant molecules and (iv) proteins that are involved in water and ion movement, such as aquaporins and ion transporters (Figure 1).

### 2.1. Transcription Factors

Transcription factors take part in the initiation of downstream genes responsive to different types of abiotic stresses. Therefore, genes encoding abiotic stress-induced transcription factors represent a valuable tool for genetic engineering experiments aimed at improving plant tolerance to different adverse environmental conditions. Transcription factors regulate gene expression through the specific binding to the *cis*-acting elements in the promoter region of the target gene. Different families of transcription factors have been shown to be involved in plant response to abiotic stresses either in abscisic acid (ABA)-dependent or ABA-independent pathways. These include (i) APETALA2/ethylene response element-binding factors (AP2/ERF), (ii) three groups of transcription factors, no apical meristem (NAM), ATAF1-2 and cup-shaped cotyledon (CUC2) that constitute a large family of transcription factors called NAC, (iii) basic leucine zipper (bZIP), (iv) WRKY and (v) MYB [12] (Figure 1).

The AP2/ERF family includes the ethylene response factor (ERF) and the dehydration-responsive element-binding protein (DREB)/C-repeat binding factor (CBF) subfamilies [13]. Transcription factors belonging to the ERF subfamily bind to the ethylene-responsive element (ERE) containing the AGCCGCC core sequence. Earlier studies showed that ERF transcription factors are mainly involved in biotic stresses, but in the last decade evidence has been reported on the role of ERFs in plant response to abiotic stresses. In *Arabidopsis thaliana*, ERF-IV regulates freezing and osmotic tolerance, whereas ERF-VII is mainly involved in response to flooding and hypoxia in *Arabidopsis* and rice [13]. DREBs/CBFs recognize dehydration-responsive or C-repeat elements (DRE/CRT) containing the A/GCCGAC core sequence on abiotic stress-responsive genes [13]. Their role as major regulators in drought, salinity, cold and heat stress has been known for a long time and has been extensively documented. In *Arabidopsis,* DREB1s play major roles in the acquisition of freezing tolerance, whereas DREB2s are mostly involved in plant tolerance to drought and heat. A role in abiotic stress tolerance has been demonstrated also for DREBs from various crops. The *ZmDREB2A* gene is involved in maize response to dehydration, salt, cold and heat stresses, the rice *OsDREB1A* and *OsDREB1B* genes are upregulated by cold, whereas the expression of *OsDREB2A* gene is induced by dehydration and high-salt stress, the *TaDREB1* gene has a role in wheat response to cold and drought, and the *HvDRF1* gene in barley is upregulated under drought, salinity or ABA treatment [13].

NACs regulate the expression of downstream target genes by binding to the NAC recognition sequence (NACRS) containing the CACG core sequence [14]. Many *NAC* genes have been shown to be involved in plant responses to various abiotic stresses. Several *Arabidopsis NAC* genes, such as *ANAC019*, *ANAC055* and *ANAC072*, are induced by drought, high salinity, cold and freezing. Similarly, in cereal crops, the rice *OsNAC6* and *OsNAC5* genes, the maize *ZmSNAC1* gene and the wheat *TaNAC69 TaNAC2a*, *TaNAC4*, *TaNAC4a* and *TaNAC6* genes are transcriptional activators involved in the response to drought, salinity and extreme temperatures [14].

Plant bZIP proteins preferentially bind to palindromic and pseudo-palindromic hexamers containing an ACGT core with flanking residues of A-box (TACGTA), C-box (GACGTC) and G-box (CACGTG). In addition, many bZIP class transcription factors have been reported that bind to the ABA-responsive element (ACGTGT/GC); these bZIPs are called ABRE binding factors (ABFs)/ABA-responsive element-binding proteins (AREBs) and regulate the plant response to abiotic stresses through the ABA-dependent signalling pathway [15]. A large number of bZIP transcription factors have been characterized and their role in the response to abiotic stresses both in model plants and crops has been reported. In *Arabidopsis,* AtbZIP17, AtbZIP24, AtbZIP60 and AtbZIP62 are involved in plant response to salt stress, whereas AtbZIP1 and AtbZIP37/AtABF3 play a role in cold and drought stress, respectively [15]. A deep characterization of the bZIP family has been carried out in rice and the function of several members has been assessed. OsbZIP05, OsbZIP12/OsABF1, OsbZIP16 and OsbZIP71 are involved in rice response to salt stress, OsbZIP20, OsbZIP42, OsbZIP46/OsABF2 and OsbZIP72 in drought, OsbZIP38, OsbZIP73 and OsbZIP87 in cold stress [15]. In wheat, a role in drought, salt and cold stress has been reported for TabZIP6, TabZIP8, TabZIP9, TabZIP13, TabZIP14-B and TabZIP60 [15].

WRKY proteins contain either one or two WRKY domains characterized by the amino acid sequence WRKYGQK and a zinc-finger-like-motif; both of these two motifs are essential for the binding of WRKYs to the TTGACT/C core sequence called W-box [16]. Numerous studies have shown that the *WRKY* genes are rapidly activated under abiotic stresses, thus ensuring an immediate and effective response to the adverse condition. In addition, a single *WRKY* gene often responds to different abiotic stresses and may, therefore, has a crucial role in the signalling process when plants are exposed to simultaneous environmental cues. AtWRKY25 and AtWRKY33 respond to both heat and salt treatments [16], whereas in wheat 15 WRKY genes have been identified, 8 of which are induced by low temperature, high temperature, NaCl and PEG treatment [17].

The MYB family is found both in plants and animals and is known to have different functions. Different MYB proteins bind to different *cis*-acting elements in the promoter of their target genes. In various plants, MYB transcription factors regulate the flavonoid pathway and are responsible for red/purple/blue pigmentation in different tissues, but they are also involved in plant growth and development and participate in the ABA-dependent signalling pathway for the regulation of the abiotic stress-responsive genes [18]. In *Arabidopsis,* AtMYB2 and AtMYB60 are involved in drought tolerance, AtMYB2 and AtMYB44 respond to salt stress, AtMYB41 and AtMYB96 are induced by both drought and salt stress, whereas AtMYB15 and MYB68 are involved in the response to low and high temperatures, respectively [18]. In rice, OsMYB2 and OsMYBS3 are involved in cold tolerance, whereas OsMYB4 has a positive effect on both cold and drought tolerance. TaMYB32, TaMYB33, TaMYB56-B and TaMYB73 are all induced by salt stress in wheat [18].

### 2.2. Osmolytes

The biochemical response of the plant cell to osmotic stress caused by dehydration is the accumulation of compatible solutes, also known as osmolytes. Osmolytes are small molecules with no toxicity at high cellular concentrations, which provide the driving gradient for water uptake, thus allowing the maintenance of cell turgor [19]. They also act in the detoxification of ROS, protection of membrane integrity, and stabilization of protein structure and for this reason, their accumulation is a widespread response to not only water stress, but also to other abiotic stresses [19]. Therefore, key enzymes involved in the metabolism of these osmolytes represent potential candidates for enhancing plant tolerance to abiotic stresses.

Osmolytes include amino acids, sugars and their derivatives [19]. Among amino acids, proline plays a central role in improving plant tolerance against various abiotic stresses. Under adverse conditions, proline accumulates in the cytosol where it contributes to the osmotic adjustment and acts as chaperone maintaining the correct protein folding and enhancing the activities of different enzymes; in addition, evidence exists suggesting its role as ROS scavenger and quencher of singlet oxygen [20]. Proline biosynthesis occurs through both the glutamate and the ornithine pathway. Glutamate is converted to proline by the sequential reactions catalyzed by the enzymes Δ^1^-pyrroline-5-carboxylate synthase (P5CS) and Δ^1^-pyrroline-5-carboxylate reductase (PYCR), whereas ornithine is converted to proline by the sequential action of ornithine aminotransferase (OAT) and PYCR [20]. Under water and salt stress conditions, proline accumulation in plants is regulated by both *P5CS* and *OAT* genes, whose expression is highly induced under these hyperosmotic stress conditions [19,21].

Glycine betaine is an amino acid derivative that accumulates in chloroplast mainly in response to water and salt stress, but also in response to cold and freezing [22]. This molecule contributes not only to the osmotic adjustment but also to the stabilization of the structure of complex proteins, such as the oxygen-evolving PSII complex and Rubisco, and the maintenance of the photosynthetic efficiency [22]. In plants, glycine betaine is synthesized from choline by two-step oxidation: the first step is catalyzed by choline monooxygenase (CMO), which converts choline to betaine aldehyde, and the second step is catalyzed by the aldehyde dehydrogenase (BADH), which converts betaine aldehyde to glycine betaine [22]. Evidence exists that salinity and drought stress increase the expression levels of the CMO and BADH encoding genes, and consequently the activity of these enzymes in the chloroplast stroma [22,23].

Sugar alcohols or polyols are sugar derivatives accumulated in high amounts in plants exposed to drought, salinity, and high and low temperatures [24]. Mannitol is an important sugar alcohol that under stress not only contributes to the osmotic adjustment but also helps in redox control through the scavenging of hydroxyl radicals [24]. In higher plants mannitol is synthesized from mannose-6-phosphate that is converted to mannitol-1-phosphate by the NADPH-mannose-6-phosphate reductase; then, mannitol-1-phosphate is dephosphorylated by the mannitol-1-phosphate phosphatase to yield mannitol [24]. The increased expression of these genes was found to be responsible for the increased accumulation of mannitol observed under drought and salt stress [25].

### 2.3. Heat Shock Proteins and Other Chaperones

Heat shock proteins (HSPs), also referred to as molecular chaperones, owe their name to the fact that they accumulate in large amounts under heat stress, but evidence exists about their induction and involvement also in the plant response to other abiotic stresses, such as drought, salinity, freezing temperature, high light, heavy metal and oxidative stress [26]. HSPs are classified into at least six different types according to their molecular weight: small HSPs (sHSPs), HSP40 (Dna), HSP60 (chaperonins), HSP70 (DnaK), HSP90 and HSP100 (Clp) [27]. HSPs are responsible for protein folding, activation, transport and degradation in a wide variety of normal cellular processes, but they are also involved in the stabilization of proteins and membranes under stress conditions to protect them from being dysfunctional, thus conferring abiotic stress tolerance [27]. In plants, the accumulation of HSPs is regulated by heat shock factors (HSFs). HSFs can activate the expression of *HSP* genes and other abiotic stress-related genes by binding to *cis*-regulatory motifs in their promoter known as heat shock elements (HSEs) [28].

Other chaperones whose expression/activity is enhanced under abiotic stress conditions include the protein disulfide isomerase and the peptidyl-prolyl *cis*/*trans* isomerase, which play an important role in the formation of disulfide bonds and in the correct folding of nascent polypeptides in the endoplasmic reticulum [29]. Experiments carried out in *Arabidopsis* have demonstrated that the accumulation of unfolded proteins, which generally occurs under abiotic stress conditions, induces the upregulation of these enzymes, thus generating a signalling pathway known as the ‘unfolded protein response’ that helps to mitigate protein damage [29]. Other chaperones localized in the endoplasmic reticulum are the calreticulin (Crt) and calnexin (Cnx); these are implicated in many cellular functions including plant response to a variety of environmental stimuli [30]. The expression of plant Crts is induced in *Arabidopsis* and wheat plants exposed to drought, in rice plants under cold stress and in *Brassica napus* plants exposed to high temperature and salt stress; conversely, the expression of Cnxs decreases in roots of soybean plants exposed to osmotic stress [30]. Evidence exists that also the chloroplast protein synthesis elongation factor (EF-Tu), a protein that plays a central role in the elongation phase of protein synthesis in the organelles of the plant cell, displays chaperone activity and protects heat-labile proteins in the chloroplast stroma from damages induced by heat stress [31].

### 2.4. Late Embryogenesis Abundant Proteins

Late embryogenesis abundant (LEA) proteins are so-called because they accumulate during the late period of seed development accompanied by dehydration. Evidence also exists that LEA proteins in vegetative organs play a role in plant response to dehydration stresses, such as drought, salinity and cold stress, probably through the maintenance of protein and membrane structure, sequestration of ions, binding of water and operation as molecular chaperones [32].

LEA proteins constitute a highly divergent group of proteins that can be classified into different groups on the basis of their amino acid sequence similarity and the presence of specific motifs [32]. While group 1 LEA proteins are found in seeds and are not involved in plant response to abiotic stresses, some members of groups 2, 3, 4, and 5 respond to different environmental cues. LEA proteins belonging to group 2, also known as dehydrins, act as molecular chaperones [32]. Two dehydrins, ERD10 and ERD14, isolated from *Arabidopsis* were found to be upregulated in response to low temperature, drought and high salinity; moreover, in vitro experiments demonstrated that ERD10 and ERD14 prevent the heat-induced aggregation of various enzymes and interact with phospholipid vesicles, thus suggesting a protective function of ERD10 and ERD14 under abiotic stress through the stabilization of proteins and membranes [33]. HVA1 is an LEA protein belonging to group 3, which accumulates in barley seeds during the late stage of seed development in response to dehydration [34]. The expression of the *hva1* gene was found to be induced by drought, salinity and cold stress, as well as by ABA treatment [35]. It has been hypothesized that HVA1 acts by sequestering ions that accumulate during dehydration and that may cause serious damage to cellular proteins and structures [34]. Similarly, LE25, a group 4 LEA protein, was found to be expressed in tomato leaves and roots in response to water stress and ABA accumulation [36].

### 2.5. ROS Detoxification

A common phenomenon in plants exposed to various abiotic stresses is the overproduction of ROS, which ultimately results in oxidative stress. Oxidative stress damages biomolecules such as lipid membranes, proteins and nucleic acids and ultimately results in plant cell death. To minimize the damaging effects of ROS, plants have evolved a complex network consisting of various enzymatic and non-enzymatic mechanisms that can reduce oxidative stress and contribute to enhancing the plant’s tolerance to various abiotic stress conditions. Among all antioxidant enzymes, superoxide dismutase (SOD) represents the first line of defence by dismutating superoxide anion into hydrogen peroxide and reducing the possibility of its conversion into hydroxyl radical by Fenton’s reaction [37]. SOD has different isoforms: the Cu/Zn-SOD isoform in the cytoplasm, chloroplasts, peroxisomes and apoplast, the Fe-SOD in the chloroplasts, and the Mn-SOD in the mitochondria and peroxisomes [37]. Hydrogen peroxide produced by SOD is detoxified to oxygen and water by catalase (CAT), glutathione peroxidase (GPX) and the ascorbate–gluthatione cycle. The ascorbate–glutathione cycle consists of the enzymes ascorbate peroxidase (APX), monodehydroascorbate reductase (MDHAR), dehydroascorbate reductase (DHAR) and glutathione reductase (GR), and the non-enzymatic antioxidants ascorbate and glutathione (GSH) [38]. In the cycle, the peroxidation of ascorbate by APX gives monodehydroascorbate. Monodehydroascorbate is either converted to ascorbate by the MDHAR or undergoes non-enzymatic disproportionation to dehydroascorbate, which is recovered and converted into ascorbate by the glutathione-dependent reaction catalyzed by the DHAR; in this latter reaction, GSH is oxidized to give glutathione dimers GSSG that are re-reduced by GR [38].

While these scavenging enzymes are important in directly regulating ROS levels, there are other enzymes, which are responsible for the repair of ROS-induced damages. These include the enzymes belonging to the superfamily of thioredoxins (TRXs) that catalyze the reduction of disulfide bonds generated under oxidative stress and restore the structure and function of proteins [39], the aldo-keto reductases that catalyze the NADPH-dependent reduction of cytotoxic aldehydes derived from lipid peroxidation, an important process that favours the reduction of stress-induced damages to biomembranes [40] and the enzymes involved in the repair of oxidative DNA damages [41].

In addition to ascorbate and GSH, other small antioxidant molecules, such as carotenoids, tocopherols and phenolic compounds, are also important for the control of ROS homeostasis in the plant cell. Carotenoids and tocopherols are the two most abundant groups of lipid-soluble antioxidants in the chloroplast, where they quench and scavenge singlet oxygen, thus protecting the photosynthetic machinery; tocopherols can also scavenge lipid peroxy radicals and yield a tocopheroxyl radical that can be re-reduced by reacting with ascorbate or other antioxidants [42]. Phenolic compounds, particularly flavonoids and phenolic acids, scavenge different free radical molecules and reduce membrane damage due to lipid peroxidation [42].

A great deal of studies has demonstrated that the activation of antioxidant enzymes and/or key enzymes involved in the biosynthesis and accumulation of small antioxidant molecules is crucial for protecting plant cells against ROS overproduction triggered by abiotic stresses both in model and crop plants. For instance, a significant increase in SOD activity occurs in tomato, chickpea and mulberry plants exposed to salt stress, and in *Phaseolus vulgaris* and rice plants exposed to water stress [43]. An increase in CAT activity occurs in wheat plants exposed to water stress and in *Cicer arietinum* plants exposed to salinity [44]. APX activity increases significantly in *Phaseolus vulgaris* and *Picea asperata* plants exposed to water stress [43], and in *Cucumis sativus* cold stress activates the enzymes of the ascorbate-glutathione cycle [44]. As for the small antioxidants, ascorbate, GSH and α-tocopherol significantly increase in tomato plants exposed to drought stress, and in soybean total phenolic and tocopherol contents increase with increasing the level of osmotic stress intensity [42]. These are just a few examples of the involvement of antioxidant enzymes and small antioxidant molecules in the abiotic stress response, but a comprehensive overview of the literature can be found in the numerous reviews on this topic [42,43,44].

### 2.6. Water Channels and Ion Transporters

Plant cell membranes contain channels for the uptake of water, also known as aquaporins, and transporters responsible for the uptake of ions. Due to their role in maintaining the plant water status, aquaporins have been deeply investigated in relation to their involvement in plant response to drought and salt stress. The most studied aquaporins are the plasma membrane intrinsic proteins (PIPs) and the tonoplast membrane intrinsic proteins (TIPs) [45]. Evidence exists that genes encoding aquaporins belonging to the PIP1 subgroup are the most responsive to drought and salt stress, undergoing a downregulation in the roots and an upregulation in the leaves, which suggests a role for these isoforms in gas exchange and stomatal opening [46,47]. In addition to the regulation of gene expression, salt stress also causes changes in the activity of aquaporins. The pH decrease observed in salt-sensitive rice cultivars maintains aquaporin in its closed state [48]. Furthermore, the reduction in cytosolic Ca^2+^ concentration caused by salt stress leads to aquaporin closure [49]. A role of aquaporins in plant response to low and high temperatures has also been reported. Evidence exists that low root temperature decreases the water uptake ability of the root [50]. Consistently, in *Arabidopsis* and rice plants exposed to low temperatures, different aquaporins of the PIP1 and PIP2 subgroups are downregulated both in the roots and the aerial part, with only the PIP2;5 aquaporins upregulated in both tissues [51,52]. These results strongly suggest a role for PIP2;5 in cold-stress acclimation through the increase in water uptake [50,52]. Upregulation of *PIP* genes occurs in tolerant cultivars of tea exposed to high temperatures, whereas a *TIP* gene is downregulated both in tolerant and sensitive cultivars [53]; also, pre-treatment of strawberry plants with sodium hydrosulfide induces systemic thermotolerance through the upregulation of *HSP* and *PIP* genes [54].

Regarding the ion transporters, a crucial role is played by Na^+^ and K^+^ transporters in the tolerance mechanisms orchestrated by the plant to counteract salt stress. In glycophytes, excessive Na^+^ is toxic for the plant cell because it determines K^+^ and Ca^2+^ deficiency and forces the plant cell to accumulate osmolytes to counterbalance the export of Na^+^ for osmotic adjustment [55]. To reduce Na^+^ levels in the cytoplasm plants may adopt two strategies: (i) exclude Na^+^ from the leaf blades, (ii) compartmentalize Na^+^ in the vacuole [55]. Na^+^ efflux outside the plant cell is mediated essentially by the Na^+^/H^+^ antiporter SOS1; physiological analyses of *sos1* mutant plants have demonstrated that SOS1 is involved in Na^+^ efflux from the cytosol to the apoplast and to the xylem from the parenchyma surrounding the vascular tissues, thus maintaining low concentrations of Na^+^ in the plant cells [56]. In addition, gene expression studies revealed that SOS1 transcript levels increase significantly in roots and to a much lesser extent in shoots of both model and crop plants exposed to high salinity [56]. The best-known transporter responsible for vacuolar Na^+^ sequestration is the Na^+^/H^+^ antiporter NHX1. Extensive studies on *Arabidopsis* and rice have demonstrated the key role played by the NHX1 antiporter in salinity tolerance, by reducing the deleterious effects of excess Na^+^ in the cytosol and maintaining osmotic balance in the vacuole by using Na^+^ as cheap osmolyte [56]. Overexpression of the *NHX1* gene significantly increases salinity tolerance in different plant species, including important crops such as tomato, rice, tobacco and wheat [56].

## 3. Improvement of Abiotic Stress Tolerance in Wheat Plants through Transgenic Approaches

Recent advances in understanding the genetic control of the mechanisms triggered by plants for counteracting abiotic stresses, including the identification and cloning of candidate genes, have encouraged private and public researchers to use these genes for engineering plants that can tolerate the adverse effects of these stresses without any negative impact on their yield. As already stated, significant progress has been achieved in the transformation of cereals, including the development of transgenic wheat lines; thus, researchers have moved from theoretical studies to introducing genes controlling traits of agronomic importance, and many of the obtained transgenic wheat lines carried better tolerance to environmental cues. Examples of the most relevant transgenic approaches aimed at improving the tolerance of wheat to drought, salinity and extreme temperatures are reported below.

### 3.1. Drought

Drought is probably the most important abiotic stress that limits crop productivity worldwide. It occurs when there is less-than-average precipitation over a prolonged period of time, with a consequent reduction of the atmospheric and soil moisture that leads to an imbalance between evapotranspiration flux and water absorption from the soil. Wheat is grown in different environments, but many of these environments have drought stress as one of the major challenges to its yield. Wheat is susceptible to drought particularly at the jointing stage when it grows rapidly and the impact of water stress can accumulate quickly, thus reducing yield potential in a relatively short period of time. In addition, exposure of wheat plants to drought stress conditions after flowering and until maturity reduces the period of grain filling and ripening, thus severely reducing yields [57].

Most of the candidate genes exploited to improve drought tolerance in wheat are transcription factors, which play a key role in signal transduction under drought stress by regulating the expression of downstream genes involved in plant response to water deficit. Transcription factors that have been successfully used for the improvement of wheat tolerance to drought mainly belong to the DREB/CBF (GmDREB1, AtDREB1, GhDREB, TaDREB3 and TaCBF5L) [58,59,60,61,62,63,64], ERF (TaERF3) [65], NAC (TaNAC69-1, SNAC1) [66,67], HD-ZipI (HaHB4) [68] and WRKY (TaWRKY2, AtWRKY30) [69,70] families, but they also include the ABA-stress-ripening (ASR) transcription factor (TaASR1-D), which is involved in drought tolerance through the ABA signalling [71], and the BES/BZR transcription factor (TaBZR2) [72] and the nuclear factor Y (NF–Y) subunit A (TaNF-YA7-5B) [73], which are known to be involved in the modulation of various physiological processes including response to abiotic stresses (Table 1). When exposed to controlled water-limited conditions these transgenic lines exhibited better growth performance and higher biomass accumulation compared to the wild-type plants. The most common responses triggered by drought in these overexpressing lines were the upregulation of ABA- and stress-responsive genes, the accumulation of compatible solutes and the activation of the antioxidant defence system, which resulted in better osmotic adjustment, higher water retention and photosynthetic efficiency, and lower ROS production and oxidative damages to plant membranes (Table 1). Interestingly, after exposure to drought stress, the overexpression of the *GmDREB1* gene also induced the expression of genes involved in the biosynthesis of melatonin and the concomitant increase in the melatonin levels in leaves and roots [59] (Table 1). In this regard, evidence exists on the role of melatonin in counteracting the deleterious effects of biotic and abiotic stresses in plants through direct scavenging of ROS and indirectly through the stimulation of plant growth regulators and the improvement of the photosynthetic and antioxidant systems [74]. Some of the wheat lines overexpressing a transcription factor were also evaluated for their tolerance to drought under field conditions. When grown under water-limited conditions in the field, the *GmDREB1* overexpressing lines exhibited better growth performances and consequently higher grain yields compared to non-transgenic plants [59] (Table 1). A field trial was also carried out for testing the *AtDREBA1* overexpressing lines that under greenhouse drought conditions presented a high survival rate and water use efficiency (WUE) [61]. Although under field conditions these transgenic lines did not outperform the wild-type plants, they presented more stable growth and yield performance across different environments [61] (Table 1). Compared to wild-type plants, wheat lines overexpressing the *HaHB4* gene grown in the open field under water-limited conditions presented better WUE and higher grain yield due to higher grain number per square meter that, in turn, was linked to higher number of spikelets per spike, tillers per plant, and fertile florets per plant [68] (Table 1). These findings indicate that transgenic approaches can be effective in improving wheat adaptability to marginal regions characterized by frequent drought events.

Significant improvement in wheat tolerance to drought has also been achieved by overexpressing genes encoding enzymes involved in the biosynthesis of osmolytes. In particular, the *Vigna aconitifolia P5CS* gene [75,76,77] and the *Arabidopsis OAT* (*AtOAT*) gene [78] have been successfully used to induce proline accumulation, the bacterial *mtlD* gene, encoding the mannitol-1-phosphate dehydrogenase and engineered for expression in higher plants [79], has been used to induce the accumulation of mannitol [80], whereas the accumulation of glycine betaine has been induced through the overexpression the bacterial *betA* gene encoding the choline dehydrogenase [81] and the *BADH* gene from *Atriplex hortensis* [82]. These overexpressing lines presented higher tolerance to drought stress as demonstrated by their higher growth rate and biomass accumulation compared to non-transgenic plants [75,76,77,78,79,80,81,82] (Table 1). Interestingly, the protective effect of these osmolytes was not always due to their involvement in the osmotic adjustment. Indeed, under water deficit, the transgenic lines overexpressing the *PC5S* gene exhibited the same pressure potential but lower levels of malondialdehyde (MDA)—an end-product of lipid peroxidation in biomembranes—and higher membrane stability compared to non-transgenic plants; this prompted the authors to hypothesize that the observed tolerance of these lines was mainly due to protection mechanisms against oxidative stress rather than to osmotic adjustment [75,76,77] (Table 1). In the same manner, the amount of mannitol accumulated in the wheat lines overexpressing the bacterial *mtlD* gene was found to be inadequate to account for osmotic effects and this suggested that the beneficial effect of mannitol was probably linked to protective mechanisms other than osmotic adjustment [80]. A different behaviour was instead observed in the wheat lines overexpressing the *betA* and the *BADH* genes. Under water deficit these lines accumulated not only glycine betaine but also other osmolytes, such as proline, soluble sugars and soluble proteins, that altogether contributed to the osmotic adjustment and determined an improvement in cell water status and stomatal opening [81,82] (Table 1). The increase in stomatal conductance together with the protective effect of glycine betaine on proteins of thylakoid membranes led to an improvement of the photosynthetic efficiency, whereas the protection of the antioxidant enzymes reduced ROS generation and oxidative damages [81,82] (Table 1).

Among those genes encoding proteins directly involved in the plant cell protection against abiotic stresses, the *HVA1* gene from barley has been used by different research groups and has proved to be particularly effective in the enhancement of wheat tolerance to drought [83,84,85,86,87] (Table 1). Under controlled water deficit conditions, the transgenic wheat lines overexpressing the *HVA1* gene had higher water retention, stomatal conductance and photosynthetic activity, as well as lower electrolyte leakage and higher membrane stability, which resulted in better growth and higher biomass accumulation than wild-type plants [83,84,85,86] (Table 1). Better performances of the *HVA1* overexpressing lines were also observed under rainfed conditions in the field, as demonstrated by the higher WUE, RWC and stable yields [87] (Table 1). It is feasible that the improvement of drought stress tolerance conferred to the wheat lines by the *HVA1* overexpression is a direct consequence of the dehydration-protective properties of LEA proteins towards membranes and macromolecules, but it may also derive indirectly from the ability of HVA1 to upregulate *NAC* and *DREB* genes that, in turn, induced the expression of genes encoding dehydrins, Rab proteins, and antioxidant proteins/enzymes, as demonstrated by the transcriptome analysis carried on these transgenic lines [85] (Table 1).

As far as the genes involved in the ROS detoxification, the wheat *TaNRX* gene [88], which encodes a nucleoredoxin belonging to the TRX family, and the *Medicago sativa MsALR* gene [89] encoding an aldose reductase belonging to the aldo-keto reductase family, were found to be effective in generating drought-tolerant wheat lines (Table 1). The overexpression of the *TaNRX* gene affected the expression of several genes including those encoding WRKY and MYB transcription factors, which are typically involved in the plant response to drought stress (Table 1). In addition, these transgenic lines exhibited higher leaf chlorophyll, proline and soluble sugar content, higher catalase, superoxide dismutase and peroxidase activities, and lower levels of MDA, hydrogen peroxide, and superoxide anion compared to the wild-type plants (Table 1). This suggests the TaNRX counteracts the oxidative stress triggered by drought both directly and indirectly through the activation of the antioxidant system. 

The overexpression of C4 photosynthetic genes in C3 plants has been widely used to improve the photosynthetic efficiency and yield of C3 plants [90]. Consistently, the wheat transformation with the maize gene encoding the phosphoenolpyruvate carboxylase (PEPC), the enzyme responsible for the primary fixation of CO_2_ in C4 and Crassulacean plants, has proven to be effective in conferring tolerance to drought stress, in which the yield loss is mainly due to the limited CO_2_ availability resulting from stomatal closure [91] (Table 1). Proteomic analysis revealed that under water stress these transgenic lines presented higher levels of proteins related to photosynthesis and plastid structural stability, higher activity of enzymes involved in the amino acid metabolism, and higher levels of cytoskeleton proteins compared to non-transgenic plants; this resulted in higher photosynthetic rate, higher accumulation of proline, glycine betaine and polyols and better growth performance (Table 1). Better growth and higher tolerance to dehydration were also observed in wheat plants overexpressing the wheat gene *TaPEPKR2* encoding the phosphoenolpyruvate carboxylase kinase-related kinase, an enzyme probably involved in the phosphorylation of the PEPC, which is essential for its activation [92] (Table 1).

In addition to the main classes of candidate genes, other genes known to be involved in the response to abiotic stresses of plants and other organisms have been exploited to enhance drought tolerance in wheat. Successful examples are the bacterial *SeCspA* and *SeCspB* genes [93], which encode cold shock proteins that protect bacteria from cold-induced damages to RNA [94], the isopentenyl transferase (*IPT*) gene from *Agrobacterium tumefaciens* that catalyzes the rate-limiting step in the cytokinin biosynthesis [95], the *Arabidopsis* SUMO cysteine protease (*OVERLY TOLERANT TO SALT-1, OTS1*) gene that is involved in the regulation of plant growth during stress [96], and the wheat ABA receptor (*TaPYL4*) gene [97] (Table 1). When exposed to drought stress, these transgenic lines presented better growth performance compared to the non-transgenic lines, as a consequence of higher water retention, higher osmolyte accumulation, better photosynthesis and upregulation of stress-related genes (Table 1). Notably, when grown under rainfed conditions in the field, the *SeCspA* and the *IPT* overexpressing lines presented higher yield and yield components, which suggested their suitability for cultivation in arid regions (Table 1).

**Table 1:** Improvement of drought tolerance in wheat plants through transgenic approaches.

Gene	Gene Product	Plant Source	Improved Traits	Ref.
Transcription factors				
*GmDREB1*	Dehydration-responsive element-binding protein	Soybean	Higher number of leaves and roots Higher soluble sugar levels	[58]
			Less membrane damage, better osmotic adjustment and photosynthetic efficiency, higher melatonin level Upregulation of stress-responsive genes (e.g., transcription factors, antioxidant enzymes, enzymes involved in the biosynthesis of melatonin) Higher yields in the field	[59]
*AtDREBA1*	Dehydration-responsive element-binding protein	*Arabidopsis* *thaliana*	Higher relative water content, higher chlorophyll, proline and soluble sugar levels	[60]
			Higher water use efficiency and biomass Stable yield performance under water-deficit conditions in the field	[61]
*GhDREB*	Dehydration-responsive element-binding protein	Cotton	Higher survival rates Higher soluble sugar level	[62]
*TaDREB3*	Dehydration-responsive element-binding protein	Bread wheat	Higher survival rates and higher yields	[63]
*TaCBF5L*	C-repeat binding factor	Bread wheat	Higher plant biomass and grain weight	[64]
*TaERF3*	Ethylene response factor	Bread wheat	Higher survival rates and lower water loss Upregulation of ABA- and stress-responsive genes (e.g., peroxidase, late embryogenesis abundant protein, ABA-responsive protein, glutathione-*S*-transferase)	[65]
*TaNAC69-1*	Protein belonging to the NAM/ATAF1-2/ CUC2 family	Bread wheat	Higher root and shoot biomass and longer roots Enhanced expression of stress-responsive genes	[66]
*SNAC1*	Protein belonging to the NAM/ATAF1-2/ CUC2 family	Rice	Higher water retention and chlorophyll content Enhanced expression of genes involved in ABA signalling (e.g., sucrose phosphate synthase, 1-phosphatidylinositol-3-phosphate 5-kinase, type 2C protein phosphatases, and regulatory components of ABA receptor)	[67]
*HaHB4*	Homeodomain-leucine zipper I protein	Sunflower	Higher water use efficiency Higher number of spikelets per spike, tillers per plant, and fertile florets per plant and higher yields	[68]
*TaWRKY2*	WRKY domain protein	Bread wheat	Higher soluble sugars, proline and chlorophyll levels and lower hydrogen peroxide levels at seedling stage Longer spike length, more kernels per spike, greater aboveground biomass, higher yields	[69]
*AtWRKY30*	WRKY domain protein	*Arabidopsis* *thaliana*	Higher shoot and root length, and biomass production Higher chlorophyll, proline and soluble sugar levels and antioxidant enzymes activities Higher photosynthetic performance and higher relative water content Lower malondialdehyde, hydrogen peroxide levels and electrolyte leakage Upregulation of stress-responsive genes (e.g., antioxidant enzymes, transcription factors and aquaporins)	[70]
*TaASR1-D*	Abscisic acid stress- ripening protein	Bread wheat	Higher survival rates and greater water retention ability	[71]
*TaBZR2*	BRI1-EMS suppressor /brassinazole-resistant family	Bread wheat	Higher survival rates, delayed leaf rolling, and proline level Lower malondialdehyde and electrolyte leakage Upregulation of abiotic stress-responsive genes	[72]
*TaNF-YA7-5B*	Nuclear factor Y transcription factors	Bread wheat	Higher shoot and root length, and biomass production Fasta stomata closing rates and reduced water losing rates Higher proline and soluble sugar levels and antioxidant enzyme activities Lower malondialdehyde and ROS levels Higher photosynthetic performance Upregulation of stress-responsive genes (e.g., Δ^1^-pyrroline-5-carboxylate synthase, superoxide dismutase and catalase)	[73]
**Osmolytes**				
*P5CS*	Δ^1^-pyrroline-5-carboxylate synthase	*Vigna* *aconitifolia*	Higher proline level, lower malondialdehyde level and higher membrane stability	[75,76,77]
*AtOAT*	Ornithine aminotransferase	*Arabidopsis* *thaliana*	Higher proline level and survival rates Upregulation of genes involved in proline biosynthesis via glutamate and ornithine pathways and downregulation of genes involved in proline catabolism	[78]
*mtlD*	Mannitol-1-phosphate dehydrogenase	*Escherichia* *coli*	Higher mannitol level, fresh weight, dry weight, plant height and flag leaf length	[80]
*betA*	Choline dehydrogenase	*Escherichia* *coli*	Higher glycine betaine, proline and soluble sugar levels Higher germination percentage and biomass, and better-developed roots Higher relative water content, and better photosynthesis Higher activity of antioxidant enzymes, lower malondialdehyde level and electrolyte leakage	[81]
*BADH*	Betaine aldehyde dehydrogenase	*Atriplex* *hortensis*	Higher glycine betaine, proline, soluble protein, soluble sugar and free amino acid levels Higher relative water content, more negative osmotic potential and higher photosynthetic efficiency Higher activity of antioxidant enzymes, lower ROS and malondialdehyde levels, and lower electrolyte leakage	[82]
**LEA proteins**				
*HVA1*	Group 3 LEA protein	Barley	Higher water use efficiency, root fresh and dry weights, shoot dry weight and total dry biomass	[83]
			Higher germination rate and root length Higher relative water content, and more negative water potential Higher stomatal conductance and photosynthetic activity Lower electrolyte leakage and higher membrane stability	[84]
			Greener leaf and more robust root growth Upregulation of drought-responsive genes (e.g., DREB and NAC transcription factors, dehydrins, ferritin, glutathione-*S*-transferase)	[85]
			Higher germination percentage, seedling growth, biomass accumulation and nitrate reductase activity at seedling stage Higher photosynthetic activity and yield at post-anthesis	[86]
			Higher water use efficiency, relative water content and stable yields in the field	[87]
**ROS detoxification**				
*TaNRX*	Thioredoxin	Bread wheat	Higher survival rates, higher chlorophyll, proline and soluble sugar levels, higher catalase, superoxide dismutase and peroxidase activities Lower malondialdehyde, hydrogen peroxide and superoxide anion levels Upregulation of genes encoding transcription factors and other stress-responsive genes	[88]
*MsALR*	Aldose reductase	*Medicago* *sativa*	Higher water use efficiency and biomass production	[89]
**Other genes**				
*PEPC*	Phosphoenolpyruvate carboxylase	Maize	Higher proline, soluble sugar and soluble protein levels Higher water use efficiency and photosynthetic rate, higher root volume and activity, biomass per plant, spike numbers per plant, grain numbers per spike and thousand grain weight, higher levels of proteins related to photosynthesis, energy metabolism, amino acid synthesis, protein synthesis and assembly, and cytoskeleton	[91]
*TaPEPKR2*	Phosphoenolpyruvate carboxylase kinase-related kinase	Bread wheat	Higher total root length	[92]
*SeCspA, SeCspB*	Cold shock proteins	*Escherichia* *coli*	Higher survival rates and proline level, and lower malondialdehyde level Upregulation of stress-responsive genes Higher yield in the field (only for *SeCspA*)	[93]
*IPT*	Isopentenyl transferase	*Agrobacterium tumefaciens*	Delayed senescence, higher yield due to a higher number of grains per spike and a higher number of spikes in the field	[95]
*OTS1*	cysteine protease (OVERLY TOLERANT TO SALT-1)	*Arabidopsis* *thaliana*	Higher growth and delayed senescence Higher relative moisture content, chlorophyll content and photosynthesis rate Lower SUMOylation of total proteins	[96]
*TaPYL4*	ABA receptor	Bread wheat	Lower stomatal opening and water loss Higher photosynthetic efficiency Higher grain yields	[97]

### 3.2. Salinity

Worldwide, the area affected by salt stress amounts to 20% of the arable area but it is gradually increasing due to climate change and anthropogenic activities [98]. Soil salinity negatively affects wheat growth from germination to harvesting; it reduces seed germination and seedling vigour by negatively affecting root length and plant height and alters many physiological and biochemical processes; this leads to a significant decline in grain yield and quality [99]. The deleterious effects of salt are due to (i) a decreased rate of water uptake into plants due to the low water potential of soil and (ii) increased uptake of toxic ions, the accumulation of which in the plant cell causes nutritional imbalance [100].

As already highlighted, drought and salt stress have similar effects on plants; so, several genes successfully exploited to improve wheat tolerance to water deficit have also been shown to be useful in inducing salt stress tolerance in this crop. These ‘multi-protecting’ genes mainly include those encoding transcription factors, as well as enzymes involved in the biosynthesis and accumulation of osmolytes. So, wheat lines overexpressing the *GmDREB* [58], *AtDREB1A* [60], *GhDREB* [62], *TaERF3* [65], *SNAC1* [67] and *TaASR1-D* [71] genes were found to be more tolerant not only to drought but also to salinity (Table 2). Improved tolerance to salt stress was also achieved by overexpressing the wheat *TabZIP15* gene [101], encoding a bZIP transcription factor, as well as the *Eutrema salsugineum EsMYB90* gene [102] and the wheat *TaMYB86B* gene [103] encoding MYB transcription factors (Table 2). When exposed to high salt levels, the physiological, biochemical and molecular mechanisms observed in all these transgenic lines were similar to those observed under drought stress conditions, that is the upregulation of ABA- and abiotic stress-responsive genes, the accumulation of osmolytes and the activation of the antioxidant enzyme system, which resulted in lower ROS accumulation and reduced oxidative damage to membranes, and better growth performance (Table 2). Interestingly, the analyses of the yield parameters revealed that the grain yield of both *TabZIP15* and *TaASR1-D* overexpressing lines was increased under salt stress conditions compared to wild-type plants, thus suggesting that these genes can be useful to breed new wheat cultivars with tolerance to high salt conditions (Table 2).

As regards the genes involved in the biosynthesis of osmolytes, increased tolerance to salinity was observed in wheat lines overexpressing the *AtOAT* [78], *mtlD* [80,104], *betA* [105] and *BADH* [106,107,108] genes (Table 2). As already observed under drought stress conditions, the overexpression of these genes under salinity contributed not only to a better osmotic adjustment but also to a better control of ROS production, which reduced damages to membranes and macromolecules and resulted in higher photosynthetic activity and better growth (Table 2). Moreover, the analysis carried out on *mtlD*, *betA* and *BADH* overexpressing lines revealed that the overproduction of osmolytes also contributed to protecting leaves from ion toxicity; indeed, transgenic lines accumulated Na^+^ and Cl^−^ in their sheaths and maintained higher levels of K^+^ in their leaves, thus reducing the leaf Na^+^/K^+^ ratio compared to non-transgenic plants (Table 2). In terms of grain yields and grain quality, the field performance of the *mtlD* and *betA* overexpressing lines in saline land areas was much better than the wild-type plants (Table 2), thus showing the promising potential of these genes in salt-tolerant wheat breeding.

A similar mechanism of tolerance to salinity was observed in wheat lines overexpressing the *HVA1* gene from barley. In addition to better seed germination, root and shoot development, lower electrolyte leakage and higher membrane stability, these lines presented lower Na^+^ levels in the shoot compared to non-transgenic plants [84] (Table 2), a phenomenon that could be linked to the ability of LEA 3 proteins to sequestrate ions under stress conditions [34].

Among the genes involved in ROS detoxification, the overexpression of the wheat peroxidase (*TaPRX-2A*) gene was found to be effective in improving wheat tolerance to salt stress [109] (Table 2). As observed under drought stress in wheat lines overexpressing the *TaNRX* gene (see Table 1), the overexpression of the *TaPRX-2A* gene exerted its positive action against salinity both directly and indirectly through the activation of other antioxidant enzymes. Indeed, the wheat lines overexpressing the *TaPRX-2A* gene showed not only higher peroxidase activity, but also higher catalase and superoxide dismutase activities, as a consequence of an upregulation of their encoding genes; this amplified the antioxidant reaction and effectively lowered the salt-induced cell oxidation, as demonstrated by the stronger reduction of ROS and MDA levels compared to non-transgenic plants (Table 2). Since TaPRX-2A was found to be located in the nucleus, it is feasible that its role under salt stress is the inhibition of ROS-mediated damage to genomic DNA, whereas the other antioxidant enzymes are responsible for ROS scavenging in other cell compartments.

A class of candidate genes typically involved in the plant response to salt stress is represented by aquaporins and ion transporters, which regulate water, and Na^+^ and K^+^ transport. Wheat lines overexpressing genes encoding aquaporins of the PIP type, such as the *SbPIP1* gene from *Salicornia bigelovii* [110], a euhalophyte that requires high Na^+^ concentration for optimal growth, and the durum wheat *TdPIP2;1* gene [111], performed much better in physiological and biochemical attributes compared to wild-type plants, showing higher osmolyte levels and antioxidant activity, as well as lower Na^+^/K^+^ ratio, which resulted in better osmotic adjustment, lower oxidative damage and better growth performance (Table 2). Interestingly, in a long-term experiment, the *TdPIP2;1* overexpressing lines reached maturity and produced filled grains (Table 2), thus suggesting they could be potentially cultivated in saline soils without major penalties for grain yield. Although the molecular basis underlying salinity tolerance in the wheat lines overexpressing the *PIP* genes was not investigated, it is feasible that the complex response observed in the *PIP* overexpressing lines is due not only to the higher PIP levels in the plasma membrane but also to PIP-induced upregulation of other stress-responsive genes, as already observed in other plant species overexpressing foreign aquaporin genes [112]. Higher salinity tolerance was also observed in the wheat lines overexpressing the *Arabidopsis AtNHX1* gene [113], which encodes the vacuolar Na^+^/H^+^ antiporter, and the barley vacuolar H^+^-pyrophosphatase (*HVP1*) gene [114], which encodes the proton pump that generates the proton gradient needed to promote Na^+^/H^+^ antiport. In both cases, the overexpressing lines presented higher germination rate and biomass accumulation compared to non-transgenic plants; moreover, when grown under saline field conditions, they also presented higher yields (Table 2). This is expected since, in addition to leaf Na^+^ exclusion, the mechanism of tissue tolerance, based on Na^+^ compartmentalization into the vacuole, represents a major mechanism of salinity tolerance in wheat [115]. Under salinity, lower Na^+^ levels were also detected in wheat plants overexpressing the bacterial *SeCspA* and *SeCspB* genes [93], and the wheat bile acid/sodium symporter 2 (*TaBASS2*) gene, responsible for the uptake into chloroplast of pyruvate, a precursor of ABA and other metabolites involved in plant response to stress [116] (Table 2). Lower Na^+^ and higher K^+^ levels were observed in wheat lines overexpressing the *TaPUB1* gene encoding a U-box E3 ubiquitin ligase, a component of the ubiquitin–proteasome pathway that regulates the activity and stability of many cellular proteins and is involved in diverse physiological processes including responses to abiotic stress [117]. When exposed to salt stress, these transgenic lines also exhibited higher proline levels and higher activities of antioxidant enzymes that contributed to a better control of ROS production compared to wild-type plants (Table 2). Transcriptional analysis revealed that these physiological responses are a consequence of the TaBUB1-induced upregulation of genes encoding ion transporters and enzymes involved in proline biosynthesis and ROS scavenging (Table 2).

**Table 2:** Improvement of salinity tolerance in wheat plants through transgenic approaches.

Gene	Gene Product	Plant Source	Improved Traits	Ref.
Transcription factors				
*GmDREB1*	Dehydration-responsive element-binding protein	Soybean	More extended leaves and plentiful roots	[58]
*AtDREBA1*	Dehydration-responsive element-binding protein	*Arabidopsis thaliana*	Higher relative water content, chlorophyll, proline and soluble sugar levels	[60]
*GhDREB*	Dehydration-responsive element-binding protein	Cotton	Higher survival rates and chlorophyll content	[62]
*TaERF3*	Ethylene response factor	Bread wheat	Higher germination and survival rates Higher chlorophyll level, lower hydrogen peroxide level and lower stomatal conductance Upregulation of ABA- and stress-sensitive genes (e.g., peroxisase, late embryogenensis abundant protein, ABA-responsive protein, glutathione-*S*-transferase)	[65]
*SNAC1*	Protein belonging to the NAM/ATAF1-2/CUC2 family	Rice	Higher survival rates and grain number Upregulation of the expression of ABA- and stress-sensitive genes and genes encoding regulatory components of ABA receptor	[67]
*TaASR1-D*	Abscisic acid stress- ripening protein	Bread wheat	Higher plant height, dry biomass, tiller number, spikelet number per spike, grain yield per plant, grain weight and grain width Lower superoxide anion, hydrogen peroxide and malondialdehyde levels	[71]
*TabZIP15*	Basic leucine zipper proteins	Bread wheat	Higher plant height, longer root length, higher aboveground and root fresh weight, longer spike length, higher number of grains per spike Lower malondialdehyde and hydrogen peroxide levels Upregulation of genes involved in metabolic processes and response to abiotic stresses	[101]
*EsMYB90*	v-myb avian myeloblastosis viral oncogene homolog family	*Eutrema salsugineum*	Higher root length and fresh weight, higher peroxidase and glutathione	[102]
*TaMYB86B*	v-myb avian myeloblastosis viral oncogene homolog family	Bread wheat	Higher biomass and K^+^ level Lower Na^+^, ROS and malondialdehyde levels, upregulation of stress-related genes	[103]
**Osmolytes**				
*AtOAT*	Ornithine aminotransferase	*Arabidopsis thaliana*	Higher proline and chlorophyll levels, and higher peroxidase and catalase activities Faster growth, higher survival rates, longer and more secondary roots and longer shoots Upregulation of genes involved in proline biosynthesis via glutamate and ornithine pathways and downregulation of genes involved in proline catabolism	[78]
*mtlD*	Mannitol-1-phosphate dehydrogenase	*Escherichia coli*	Higher mannitol levels Higher shoot fresh weight, dry weight, plant height and flag leaf length	[80]
			Higher proline, mannitol, soluble sugar, chlorophyll and K^+^ levels, and higher activities of enzymatic and non-enzymatic antioxidants Higher number of leaves and leaf area per plant, root system size and plant dry weight Higher number of spikes and grain weight per plant, and thousand grain weight Higher grain content of starch, protein and soluble sugars	[104]
*betA*	Choline dehydrogenase	*Escherichia coli*	Higher glycine betaine, proline and soluble sugar levels Higher relative water content and more negative osmotic potential Lower Na^+^/K^+^ ratio, malondialdehyde level and electrolyte leakage Higher germination rates, more tillers and higher grain yields in the field	[105]
*BADH*	Betaine aldehyde dehydrogenase	*Atriplex* *hortensis*	Higher glycine betaine, proline, and soluble protein and sugar levels, and higher activity of antioxidant enzymes Better osmotic adjustment, lower Na^+^ and higher K^+^ levels in the leaves Lower ROS and malondialdehyde levels, and lower electrolyte leakage	[106]
			Higher glycine betaine, chlorophyll and carotenoid levels Modification of the lipid composition of thylakoid membranes and higher photosynthetic activity	[107]
*HvBADH1*	Betaine aldehyde dehydrogenase	Barley	Higher glycine betaine and K^+^ levels Higher survival rates	[108]
**LEA proteins**				
*HVA1*	Group 3 LEA protein	Barley	Higher germination rate and root length Lower electrolyte leakage and higher membrane stability Lower Na^+^/K^+^ ratio in the shoot	[84]
**ROS detoxification**				
*TaPRX-2A*	Peroxidase	Bread wheat	Higher survival rates and shoot length Higher relative water content Higher proline, soluble sugar and soluble protein levels Higher peroxidase, catalase and superoxide dismutase activities Lower malondialdehyde, superoxide anion and hydrogen peroxide levels Upregulation of ABA- and stress-responsive genes (e.g., ROS scavenging enzymes, thumatin-like protein, glutathione *S*-transferase)	[109]
**Aquaporins and** **ion transporters**				
*SbPIP1*	Plasma membrane intrinsic proteins	*Salicornia* *bigelovii*	Higher proline and soluble sugar levels and lower malondialdehyde level	[110]
*TdPIP2;1*	Plasma membrane intrinsic proteins	Durum wheat	Higher catalase and superoxide dismutase activities, and lower malondialdehyde and hydrogen peroxide levels Lower Na^+^ level and higher K^+^ level in the shoots Higher germination rate, higher biomass and filled grains	[111]
*AtNHX1*	Vacuolar Na^+^/H^+^ antiporter	*Arabidopsis thaliana*	Lower Na^+^ and higher K^+^ levels in the leaves Higher germination rates, biomass production, and heavier and larger grains in the field	[113]
*HVP1*	Vacuolar pyrophosphatase	Barley	Higher photosynthesis rate, stomatal conductance, transpiration rate and water use efficiency Higher germination rate, plant height, spike length, number of spikelets per spike, 1000 grain weight, grain yield and harvest index in the field	[114]
**Other genes**				
*SeCspA, SeCspB*	Cold shock proteins	*Escherichia coli*	Higher fresh weight and lower Na^+^ content	[93]
*TaBASS2*	Pyruvate transporter	Bread wheat	Lower Na^+^ level and ROS scavenging	[116]
*TaPUB1*	U-box E3 ubiquitin ligase	Bread wheat	Longer shoot and root Higher chlorophyll, proline and soluble sugar levels Higher photosynthetic rate, transpiration rate and stomatal conductance Higher catalase, superoxide dismutase and peroxidase activities Lower malondialdehyde, superoxide anion and hydrogen peroxide levels Lower Na^+^ and higher K^+^ levels in the root Upregulation of stress-responsive genes (e.g., ion transporters, antioxidant enzymes and enzymes involved in proline biosynthesis)	[117]

### 3.3. High Temperatures

Climate changes are causing a progressive increase in the earth’s temperature and this phenomenon represents a serious threat to crop yields worldwide. Plants experience heat stress when they are exposed to temperatures above a certain threshold level for long enough to cause irreversible damage to their growth and productivity [118]. Wheat can be subjected to heat stress conditions throughout its growth cycle; however, the greatest damages occur when high temperatures coincide with the reproductive and grain filling stages of this crop. The persistence of high temperatures during these stages reduces both grain yield and quality. It has been estimated that for each 1 °C increase above the optimum temperature range of 15–20 °C for wheat, the grain filling duration decreases on average by 2.8 days [119] and the grain yield is reduced by 6% [120].

Wheat lines transformed with the *AtWRKY30* gene were found to be resistant not only to drought but also to heat stress [70] (Table 3). The *AtWRKY30* overexpression enhanced wheat tolerance to heat stress via inducing the same molecular, physiological and biochemical responses observed under drought stress, that is the induction of osmolyte biosynthesis, gas exchange parameters, antioxidant enzyme activity and expression of stress-related genes (Table 3). This is expected since for most crops including wheat water and heat stress often occur simultaneously and induce plants to activate the same defence mechanisms to deal with both these stresses [121]. Other transcription factors successfully used to improve heat tolerance in wheat are the HSFs, which regulate the expression of the *HSP* genes. This is a typical plant response to prevent heat-induced protein misfolding and dysfunction [122]. Evidence has been reported that in wheat plants exposed to high temperatures the HSFA2 and HSFA6 members become the dominant HSFs, thus suggesting an important regulatory role of these transcription factors during heat stress [123]. Consistently, transgenic wheat lines overexpressing the wheat *TaHsfC2a-B* and *TaHsfA6f* genes exhibited higher tolerance to high temperatures compared to non-transgenic plants, as demonstrated by their longer shoot and root, and higher biomass accumulation [124,125] (Table 3). Expression analysis of these transgenic lines revealed that both TaHsfC2a-B and TaHsfA6f are two important regulators of wheat adaptation to heat stress that act by inducing the expression of several *HSP* genes and other genes involved in heat stress tolerance (Table 3). As said above, another protein able to act as a chaperone and protect the photosynthetic-related enzymes from damage induced by heat stress is EF-Tu [31]. Consistently, reduced thermal aggregation of leaf proteins, reduced damage to thylakoid membranes and ultimately higher yields were observed in transgenic wheat lines overexpressing the maize *Zmeftu1* gene [126,127] (Table 3).

Consistent with the observation that common signalling events exist that are common to more than one stress type, several genes used to increase the tolerance of wheat to drought and/or salt stress have also been shown to be effective in increasing tolerance to high temperatures. These include the *AtOAT* gene [78] and the *BADH* gene from *Atriplex hortensis* [82] involved in the accumulation of osmolytes, the *HVA1* gene from barley [85], and the *ZmPEPC* [128] and the Ta*PEPKR2* [92] genes involved in the CO_2_ fixation in C4 and Crassulacean plants. However, in addition to responses similar to other abiotic stresses, specific responses to heat stress were also observed in these transgenic lines. Indeed, as already observed under water and salt stress, heat-stressed wheat lines overexpressing the *AtOAT* gene exhibited the activation of the glutamate pathway for proline biosynthesis, but unlike the other two stress conditions, heat stress did not induce proline biosynthesis via the ornithine pathway, and this was probably the reason why tolerance to high temperatures was only partial [78] (Table 3). Furthermore, the accumulation of glycine betaine due to the overexpression of the BADH gene from *Atriplex hortensis* counteracted the heat stress by improving the photosynthetic capacity, as already observed under drought stress; but whereas the improvement of photosynthesis observed under drought stress was due to an osmotic adjustment, under heat stress it was mainly due to the activation of the antioxidant system, which reduced the accumulation of ROS and the peroxidation of membrane lipids [82] (Table 3). Similarly, in the wheat lines overexpressing the *HVA1* gene, the response triggered by exposure to heat stress was mainly directed towards the control of ROS production (Table 3) rather than to the increase in water retention, as observed when these transgenic lines were exposed to drought (see Table 1). A possible explanation emerges from the transcriptomic analysis. Indeed, while drought stress induced the expression of *DREB* and *NAC* genes (see Table 1), exposure to a high temperature determined the upregulation of *HPS* and *HSF* genes (Table 3). As observed under drought stress conditions, wheat lines overexpressing the *ZmPEPC* gene, when exposed to high temperature, showed a higher photosynthetic rate and better growth performance compared to non-transgenic plants (Table 3). Consistently, transcriptomic analysis on heat-stressed lines revealed the upregulation of photosynthesis-related genes (Table 3), which is in line with the higher levels of photosynthesis-related proteins observed in the same lines exposed to drought stress (see Table 1). Moreover, under heat stress, these transgenic lines also presented the higher activity of antioxidant enzymes, which resulted in lower ROS levels and reduced oxidative damage (Table 3).

**Table 3:** Improvement of heat tolerance in wheat plants through transgenic approaches.

Gene	Gene Product	Plant Source	Improved Traits	Ref.
Transcription factors				
*AtWRKY30*	WRKY domain protein	*Arabidopsis* *thaliana*	Higher shoot and root length, and biomass production Higher chlorophyll, proline and soluble sugar levels, and antioxidant enzymes activities Higher photosynthetic performance and higher relative water content Lower malondialdehyde and hydrogen peroxide levels, and electrolyte leakage Upregulation of stress-responsive genes (e.g., antioxidant enzyme, transcription factors and aquaporins)	[70]
*TaHsfC2a-B*	Heat shock factor	Bread wheat	Higher survival rates, shoot and root length and dry biomass Higher chlorophyll content and lower electrolyte leakage Upregulation of heat shock protein genes and other ABA- and stress-responsive genes (e.g., galactinol synthase, heat-stress-associated 32-KD protein, α-amylase, filamentation temperature sensitive family metalloprotease and calcium-binding EF-hand family protein)	[124]
*TaHsfA6f*	Heat shock factor	Bread wheat	Longer shoot and higher number of roots Upregulation of heat shock protein genes and other stress-responsive genes (e.g., Rubisco activase large isoform, Golgi anti-apoptotic protein and glutathione-S-transferase)	[125]
***Chaperones***				
*Zmeftu1*	Elongation Factor thermo-unstable	Maize	Lower thermal aggregation of leaf proteins and heat injury to thylakoid membranes Higher rate of CO_2_ fixation	[126]
			Higher number of grains per plant, total grain mass per plant, and single grain mass	[127]
**Osmolytes**				
*AtOAT*	Ornithine aminotransferase	*Arabidopsis* *thaliana*	Higher proline level Upregulation of genes involved in proline biosynthesis via glutamate pathway and downregulation of genes involved in proline catabolism	[78]
*BADH*	Betaine aldehyde dehydrogenase	*Atriplex* *hortensis*	Higher glycine betaine level Higher catalase, superoxide dismutase and peroxidase activities Lower hydrogen peroxide, superoxide anion and malondialdehyde levels	[82]
**LEA proteins**				
*HVA1*	Group 3 Late Embryogenesis Abundant protein	Barley	Lower superoxide anion and hydrogen peroxide levels Larger spikes and grain size, and higher grain weight Upregulation of stress-responsive genes (e.g., *HsfA6* transcription factor, HSPs, glutathione-S-transferase, ferrodoxin, ABA-induced plasma membrane protein PM19, caleosin, cytochrome P450 and haem peroxidase)	[85]
**ROS detoxification**				
*TaFER-5B*	Ferritin	Bread wheat	Lower ROS levels and membrane damages Higher photosynthetic activity	[129]
**Other genes**				
*ZmPEPC*	Phosphoenolpyruvate carboxylase	Maize	Higher chlorophyll levels, photosynthetic rate, superoxide dismutase, catalase and peroxidase activities Lower superoxide anion, hydrogen peroxide and malondialdehyde levels Upregulation of photosynthesis-related genes (e.g., phosphoenolpyruvate carboxykinase, fructose bisphosphatase and triose phosphate translocator)	[128]
*TaPEPKR2*	Phosphoenolpyruvate carboxylase kinase-related kinase	Bread wheat	Lower wilting Lower electrolyte leakage	[92]
*SSI*	Soluble starch synthase I	Rice	Longer grain filling period Higher thousand grain weight	[130]

Better control of ROS production was also observed in wheat lines overexpressing the wheat ferritin *TaFER-5B* gene [129] (Table 3). This is probably linked to the ability of ferritin to transform toxic Fe^2+^ to the non-toxic chelate complex, thus conferring protection to cells against the oxidative stress triggered by plant exposure to high temperatures. Consistently, a reduced stress-induced membrane injury and better photosynthetic activity characterized these transgenic lines compared to the wild-type ones (Table 3).

The wheat starch synthase (SS) is a thermo-labile enzyme, and its heat inactivation has been found to limit starch deposition in wheat grains [130]. Moreover, evidence has been reported that the expression of the wheat *SS* gene is downregulated under heat stress [131]. In light of this, the rice *SSI* gene, which is heat stable at temperatures up to 35 °C, has been exploited to enhance the wheat yield under heat stress [132] (Table 3). Heat-stressed transgenic wheat lines had an increased grain filling duration and significantly higher thousand kernel weight compared to non-transgenic plants, likely due to higher starch deposition under high temperatures (Table 3). The authors hypothesized that the longer grain filling period observed in transgenic lines was the consequence of a greater translocation of sugars from leaf to seed, which is known to reduce the feedback inhibition of leaf sugar on photosynthesis [133].

### 3.4. Low Temperatures

Wheat plants are most sensitive to low temperatures during the reproductive stage when a sudden overnight drop of temperatures only a few degrees below 0° C can damage the sensitive reproductive tissues, thus resulting in spike (partial) sterility and significant yield losses [134]. In its vegetative stages, wheat can tolerate freezing temperatures up to −20 °C through cold acclimation after being exposed for a prolonged period to low temperatures between 0 and 5 °C [135]. The acquisition of freezing tolerance is carried out through many transcriptional and biochemical changes, including the activation of cold-regulated genes, the modification of membrane lipid composition, the accumulation of osmolytes and other protective and antifreeze proteins [135].

Like other abiotic stresses, tolerance to low temperatures has been achieved by overexpressing genes encoding transcription factors and enzymes involved in the biosynthesis of osmolytes. Indeed, improved tolerance to freezing was observed in transgenic wheat lines overexpressing the cotton *GhDREB* gene [62] and the *BADH* gene from *Atriplex hortensis* [136] (Table 4). When exposed to freezing temperatures, the *GhDREB* transgenic lines grew normally, whereas the growth of wild-type plants was retarded, with survival rates significantly higher in the former compared to the latter (Table 4). As already observed for the other stresses, transgenic lines overexpressing the *BADH* gene and exposed to cold stress exhibited higher levels of glycine betaine, proline and soluble sugars [136] (Table 4), which may all function as cryoprotectants by helping to protect membrane proteins and enzymes from cold-induced damages. Consistently, the cold-stressed transgenic lines maintained better membrane integrity and functionality compared to wild-type plants, as demonstrated by the lower electrolyte leakage and the higher activity of the plasma membrane H^+^-ATPase (Table 4). Under cold stress, these transgenic lines also presented lower ROS production and membrane lipid peroxidation compared to non-transgenic plants [136] (Table 4). This may be ascribable both to the ability of osmolytes to act as ROS scavengers and to protect the structure and the activity of the antioxidant enzymes, as demonstrated by the higher catalase and peroxidase activities detected under cold stress in the *BADH* overexpressing lines compared to wild-type plants (Table 4).

The protection of plant membranes from cold-induced damage has been achieved also by overexpressing the *BLT101* gene from barley [137] (Table 4). This gene encodes a lipid transfer protein (LTP) able to modulate the local lipid composition and fluidity of plant membranes [138] and is upregulated in barley plants exposed to cold stress [139]. Consistently, wheat plants overexpressing the barley *BLT101* gene exhibited reduced leakage of intracellular substances and enhanced freezing tolerance compared to the wild-type plants; in addition, the transgenic lines that underwent cold acclimation maintained higher water content compared to wild-type plants (Table 4).

## 4. Improvement of Abiotic Stress Tolerance in Wheat Plants through Genome Editing Approaches

The excitement grew with the advancements in genome editing tools, particularly the CRISPR/Cas9 system, which opened new opportunities for precise and efficient target modification of desired genes aimed at improving traits of agronomic importance in crops. However, to date, the use of genome editing approaches for the mitigation of the adverse effects of abiotic stresses in cereal crops has been rather limited and has mainly concerned rice and in very few cases wheat. The main reason is the polyploid nature of wheat, which makes its genome very complex, and ’buffered’ with respect to the effects of mutations. In polyploid genomes, some mutations, such as the knockout of genes, are generally inefficient and often do not result in any subtle changes in the phenotype due to the compensation by homoeologous copies of the edited gene. Therefore, efficient manipulation of the desired trait in these crops would require the editing of all the homoeologs, thus reducing the effectiveness of editing approaches. On the other hand, CRISPR/Cas9 is a valid tool for targeted mutagenesis in polyploid species, as multiple copies of the same gene sharing a high level of sequence similarity can be targeted simultaneously by using a common sgRNA; alternatively, when the level of similarity between genes is not particularly high, multiple sgRNAs, each targeting a single gene, can be delivered simultaneously [140]. By using CRISPR/Cas9, many important wheat traits, such as disease resistance, grain yield and quality, pre-harvest sprouting, and plant architecture have been so far improved [141].

As for the tolerance to abiotic stresses, only a few studies have been carried out to date on wheat in which the CRISPR/Cas9-mediated knockout approach has been used to validate the involvement of putative stress-responsive genes in the wheat response to the stress signal. The CRISPR/Cas9 genome editing method has been successfully applied to carry out the targeted editing of two stress-responsive genes encoding the transcription factors TaDREB2 and TaERF3 in wheat protoplasts [142]. These genes were chosen because they were found to be upregulated in wheat seedlings exposed to drought stress [142]. For both genes, a single sgRNA was designed that caused alterations in two of the three homeologs, whereas the third copy the of *TaDREB2* and *TaERF3* genes was not edited because of two and one mismatch, respectively, between the gene and the designed sgRNA [142]. CRISPR/Cas9 has also been used to validate the role of the histone acetyltransferase TaHAG1 in wheat tolerance to salinity [143]. TaHAG1 was found to contribute to salinity tolerance by modulating ROS generation; in wheat plants exposed to salt stress TaHAG1 increased the H3 acetylation and the transcriptional upregulation of a subset of genes involved in the production of hydrogen peroxide [143]. A single sgRNA was designed to target a highly conserved region in the first exon of the *TaHAG1* gene. No homozygous mutant lines with the simultaneous knockout of the three *TaHAG1* homeologs were obtained, which suggested that the homozygous mutation in all three homeologs of *TaHAG1* may be lethal for wheat; conversely, wheat lines with the simultaneous homozygous mutations at two *TaHAG1* homeologs were identified, which showed more sensitivity to salt stress as compared to wild-type plants, with significantly reduced spike length, spike kernel number and grain yield [143]. The validation of the role of the Multiprotein Binding Factor 1 (MBF1) in the tolerance of wheat to heat stress has been also carried out by using CRISPR/Cas9 [144]. MBF1 is a transcriptional co-activator that mediates transcriptional activation by interconnecting the transcription factor with the TATA-box binding protein; it participates in the regulation of different developmental processes and its role in thermotolerance has been demonstrated in *Arabidopsis* [145]. The authors applied the CRISPR/Cas9-based gene editing to target the three homeologs of the *TaMBF1c* gene that were knocked out simultaneously. Mutant wheat lines exhibited significantly decreased heat tolerance compared to wild-type plants [144].

Altogether, these preliminary findings suggest that the CRISPR/Cas9 system is an efficient tool for targeted genome editing in wheat and it has a potential application for the manipulation of wheat genome aimed at generating new wheat lines with better crop performances under adverse environmental conditions.

## 5. Conclusions

As one of the major food sources worldwide, protecting wheat from the deleterious effects of abiotic stresses is crucial to keeping its supply at adequate levels for future generations. In this context, genetic engineering showed immense potential to solve the problem of yield losses due to climate changes. Genetic transformation has proved to be a powerful tool to introduce foreign genes into plants and its application to wheat has been greatly improved. Researchers have moved from the use of genetic transformation of model plants for the assessment of the role and function of stress-responsive genes to its application for the generation of new cultivars with improved stress tolerance. However, despite the efficiency of wheat transformation has been significantly improved, transgenesis is still not routinely applied to wheat breeding; in addition, most of the transgenic wheat lines obtained have only been evaluated under greenhouse conditions, while information about their performances in the open field is very limited. Future studies are needed to fill these gaps and open the possibility for the routine introduction of exogenous genes in elite cultivars to improve traits of agronomic interest, including tolerance to abiotic stresses. The emerging opportunity of gene editing, especially with the use of the CRISPR/Cas9 system, will lead to rapid advances in wheat breeding by introducing targeted modifications directly into a cultivar of interest. After the advent of next-generation sequencing technologies, the complete genomes of both hexaploid and tetraploid wheat have been sequenced and are now publicly available. This vast information will greatly facilitate the identification of homoeologous target sites suitable for gene editing, and the editing approach aimed at increasing the tolerance of wheat to abiotic stresses will undoubtfully make progress in the near future. Altogether, these biotechnological approaches, transgenic and transgene-free, coupled with high-throughput plant genotyping and phenotyping, will enable the development of rapid and precise breeding programs that will be crucial to meet the food needs of the growing world population.

## Figures and Tables

**Table 4:** Improvement of cold and freezing tolerance in wheat plants through transgenic approaches.

Gene	Gene Product	Plant Source	Improved Traits	Ref.
Transcription factors				
*GhDREB*	Dehydration-responsive element-binding protein	Cotton	Higher survival rates	[62]
**Osmolytes**				
*BADH*	Betaine aldehyde dehydrogenase	*Atriplex* *hortensis*	Higher levels of glycine betaine, proline and soluble sugars Lower electrolyte leakage and higher plasma membrane H^+^-ATPase activity Higher catalase and peroxidase activity Lower hydrogen peroxide, superoxide anion and malondialdehyde levels	[136]
**Other genes**				
*BLT101*	Lipid transfer protein	Barley	Lower leakage of intracellular substances under freezing temperatures Lower water loss under cold acclimation	[137]
